# Factors influencing college teachers’ adoption of live online teaching: a conditional process model of technology acceptance, user satisfaction and privacy concerns

**DOI:** 10.3389/fpsyg.2023.1293879

**Published:** 2024-01-10

**Authors:** Wan Xiao, Meiqin Wang, Jiaqian Mo

**Affiliations:** Department of Educational Technology, College of Education Science and Technology, Nanjing University of Posts and Telecommunications, Nanjing, China

**Keywords:** live online teaching, technology acceptance, user satisfaction, privacy concerns, online education

## Abstract

**Purpose:**

In recent times, live online teaching has emerged as a prominent trend in online education. However, teachers are confronted with the challenge of not only acquainting themselves with the associated technologies but also effectively integrating them into their teaching practices. This dual challenge exerts pressure on teachers to adopt live online teaching. This study aims to explore the factors and mechanisms influencing teachers’ attitudes and continuance intention toward live online teaching. It covers both intrinsic and extrinsic motivations, as well as both enabling and inhibiting factors, thus providing valuable suggestions for encouraging teachers to engage in live online teaching actively.

**Method:**

This study proposed a conceptual model based on the Technology Acceptance Model, Uses and Gratifications Theory, and Communication Privacy Management Theory. A simple random sampling method was employed to recruit participants from a university in eastern China. With 224 college teachers participating in the study, various analyses, including descriptive analysis, regression analysis, and simple slope analysis, were conducted to explore the factors and mechanisms influencing college teachers’ adoption of live online teaching.

**Results:**

The study revealed the following key findings: (a) perceived easy of use had a positive impact on perceived usefulness and user satisfaction; (b) perceived usefulness had a positive effect on user satisfaction; (c) both perceived usefulness and user satisfaction positively influenced teachers’ adoption of live online teaching; (d) perceived easy of use did not directly affect teachers’ adoption of live online teaching; (e) privacy concerns exhibited a moderated effect on the relationship between perceived easy of use and perceived usefulness, as well as the relationship between perceived easy of use and user satisfaction.

**Conclusion:**

The study reveals a conditional process model elucidating teachers’ adoption of live online learning. The model incorporates perceived ease of use as a predictor, perceived usefulness and user satisfaction as two mediators, and private concerns as a moderator. The findings suggest that stakeholders should collaborate closely to enhance the design and development of the live online teaching platforms. Additionally, efforts should be made to support and improve teachers’ information literacy, fostering their enthusiasm and facilitating their professional development in live online teaching practice.

## 1 Introduction

Over the past two decades, education has undergone a significant transformation, with digital technology now being extensively utilized for teaching, learning, and assessment (Dwivedi et al., 2022). Online teaching and learning have been integral to higher education for nearly two decades (Singh and Thurman, 2019). The COVID-19 pandemic further catalyzed a rapid transition to online teaching and learning for most higher education institutions worldwide (UNESCO and IESALC, 2020). Live online teaching, involving real-time teaching activities conducted through live broadcasting (Abdous and Yen, 2010), has received widespread attention from educators and researchers. It facilitates immediate and direct communication between teachers and students, fostering active engagement and collaboration through real-time discussions, questions, answers, and feedback. While playing a crucial role during the COVID-19 pandemic, live online teaching has also emerged as a flexible teaching approach.

Despite its advantages, live online teaching poses challenges for educators, requiring them not only to acquire technical skills but also to integrate technologies into teaching meaningfully (Chou and Chou, 2021). Existing research indicates that various factors determine the success or failure of technology integration (Straub, 2009). Firstly, teachers need to be proficient in various functions of live online teaching platforms to meet the diverse demands of synchronous teaching (Hassan et al., 2020). Additionally, pedagogical practices must adapt to effectively integrate technology into teaching (Bailey and Card, 2009). Online instructors face the task of creating, developing, and managing online courses, maintaining their own identities and attributes as instructors, assessing and evaluating learning outcomes, and communicating effectively with learners in the absence of physical presence and interaction (Alman et al., 2012; Palloff and Pratt, 2013; Albrahim, 2020). It is also important for teachers to consider time management issues since there are different patterns of sense and control of time in online courses (Alman et al., 2012). Meanwhile, concerns related to plagiarism, copyright, and privacy also contribute to the challenges faced by teachers in online teaching (Hassan et al., 2020; Chou and Chou, 2021). Consequently, some teachers maintain a conservative attitude toward online teaching or even refused to use online teaching tools (Tang et al., 2021). However, teachers’ attitude and their participatory role in online classes significantly impact students’ learning motivation (Mohamad et al., 2015) and have important influence in the success of online teaching practices (Baran, 2011).

Addressing these challenges and concerns is crucial for promoting teachers’ engagement in live online teaching. Drawing on the Theory of Planned Behavior, which posits that individuals’ attitudes consciously influence their behavior, a positive attitude is anticipated to yield a positive impact on their behavioral intention and investment (Fishbein and Ajzen, 1975). In fact, existing research has also demonstrated a positive relationship between teachers’ attitude and their intention to use online technologies, such as online education platforms, digital games, metaverse-based learning environment, chatbots, and so on (Chou and Chou, 2021; Yeo et al., 2022; Zhou et al., 2022; Al-Adwan et al., 2023; Chocarro et al., 2023). Moreover, detailed variables, such as perceived usefulness, perceived ease of use, technology features, environmental support, satisfaction, self-efficacy, anxiety, social norms, and subjective features like age, gender, education background, teaching experience, job relevance, etc., have been examined to explain teachers’ adoption of educational technologies directly or indirectly (Ibili et al., 2019; Scherer et al., 2019; Hong et al., 2021; Yeo et al., 2022; Chocarro et al., 2023). However, the factors and mechanisms impacting teachers’ adoption of live online teaching still remains unclear, necessitating a context-based comprehension of this particular technology (Zhang et al., 2022). Furthermore, the majority of research tends to concentrate on enabling perceptions while neglecting inhibiting perceptions like perceived stressful, privacy risk, etc. Addressing these inhibiting factors may contribute to more effective technology adoption by exploring the factors that drive resistance (Kim and Kankanhalli, 2009; Al-Adwan et al., 2023). Therefore, the aim of this study is to investigate the main factors that facilitate and impede college teachers’ adoption of live online teaching and explore how these factors interact with each other. To achieve this aim, this study proposes a conceptual model that integrates Technology Acceptance Model, Uses and Gratifications Theory, and Communication Privacy Management Theory. The model incorporates technology acceptance, user satisfaction, and privacy concerns, covering both intrinsic and extrinsic motivations, as well as both enabling and inhibiting factors. By providing valuable insights into these factors, this study aims to advance current knowledge in the field and offer suggestions to motivate teachers actively to participate in live online teaching.

## 2 Theoretical foundation and research model

This study aims to explore the factors underlying teachers’ adoption of live online teaching. Therefore, we utilize the Technology Acceptance Model, which is one of the most well-known models for predicting technology adoption, as the foundation theory. This theory reveals the key extrinsic motivations from the technology side. Additionally, since live online teaching is a form of media technology, the Uses and Gratifications Theory elucidates the significant intrinsic motivations driving individuals to use a specific technology. Besides, in this research, our focus extends to both enabling and inhibiting factors that impact teachers’ adoption of live online teaching. Considering that teachers’ personal information, such as voice and face, is exposed synchronously to online students in live online learning environment, protecting teachers’ privacy and ensuring data security is an extremely important matter. However, privacy breaches occasionally occur in real live online teaching situation (Chou and Chou, 2021). Therefore, we incorporate the Communication Privacy Management Theory into our research model to examine how privacy concerns impact teachers’ adoption of live online teaching.

### 2.1 Technology acceptance model

To explain and predict a person’s intentions and actual use of a specific technology, Fred Davis adapted the Theory of Reasoned Action (TRA) and proposed the Technology Acceptance Model (TAM) (Davis, 1986), which has emerged as a leading scientific paradigm for investigating the acceptance of educational technology by students, teachers and other stakeholders. TAM suggests that a person’s intention can be explained by two main factors, namely perceived usefulness (PU) and perceived ease of use (PEOU). Perceived usefulness is defined as the degree to which the person believes that using the technology would enhance their performance, whereas perceived ease of use is defined as the degree to which the person believes that using the technology would be free of effort (Davis, 1986). According to TAM, perceived usefulness and perceived ease of use directly influence behavioral intention and have been proven to be antecedent factors affecting the acceptance of educational technology (Granić and Marangunić, 2019; Mazman Akar, 2019; Al-Adwan, 2020; Shen et al., 2022). Furthermore, various researchers have reported a positive relationship between perceived ease of use and perceived usefulness (Davis, 1989; Joo and Sang, 2013; Nagy, 2018). Specifically, individuals generally tend to perceive technologies as useful when they are easy to use. In contrast, if the technologies are complex, complicated, or difficult to use, individuals are less likely to perceive their usefulness (Camilleri and Falzon, 2021; Mailizar et al., 2021). Hence, we hypothesize that:

*H1*: Teachers’ perceived ease of use has a positive influence on their perceived usefulness toward live online teaching.

*H2*: Teachers’ perceived ease of use has a positive influence on their adoption of live online teaching.

*H3*: Teachers’ perceived usefulness has a positive influence on their adoption of live online teaching.

### 2.2 Uses and gratifications theory

User satisfaction is generally regarded as users’ attitude toward the object, classified as “object-based attitude” (Wixom and Todd, 2005). According to the Uses and Gratifications Theory, individuals actively and subjectively select and engage with media based on their specific needs, motivations, and goals. It recognizes individuals as active agents who choose media to fulfill their own gratifications (Katz and Foulkes, 1962). Therefore, during the process of media selection and engagement, individuals conduct a series of media evaluations. Before choosing whether to engage with a particular media technology, individuals consider its usefulness and ability to fulfill their own needs (ie. perceived usefulness), and then assess the ease of accessing and using the media technology (ie. perceived ease of use). The initial media impression may impact individuals’ attitude toward media technology, influencing their subsequent intention and behavior toward continued media technology usage (Katz et al., 1974). Based on this theory, teachers, when considering whether to adopt live online teaching, will first take into account the usefulness and ease of use, with teachers’ user satisfaction toward live online teaching playing a mediating role during this process. In the education field, research has shown that individuals are inclined to form a positive view of a specific technology and exhibit a heightened intention to use it when they experience satisfaction (Esteban-Millat et al., 2018; Faqih and Jaradat, 2021; Zhou et al., 2022). Therefore, this study proposes the following hypotheses:

*H4*: Teachers’ perceived easy of use positively affect their user satisfaction toward live online teaching.

*H5*: Teachers’ perceived usefulness positively affect their user satisfaction toward live online teaching.

*H6*: Teachers’ user satisfaction positively affect their adoption of live online teaching.

### 2.3 Communication privacy management theory

Privacy encompasses an individual’s capacity to control others’ access to their personal information (Kamal and Radhakrishnan, 2019). Privacy concerns refer to individuals’ apprehensions about potential privacy loss due to information disclosure to specific external agents or institutions (Xu et al., 2011). According to the Communication Privacy Management Theory (CPM), individuals possess a sense of ownership over their private information and actively make decisions about what information to disclose, to whom, and under what circumstances. These decisions are guided by the establishment of privacy boundaries, delineating the limits of what is considered private and who has access to that information. Additionally, CPM emphasizes that privacy is not a static state but rather a dynamic process that involves negotiating and coordinating privacy rules (Petronio, 2002).

With the rapid development of educational technologies, the exchange of individual information has become more convenient. However, many educators and students are worried about privacy issues in the online learning environment (Peng and Dutta, 2022), impacting their attitudes toward adopting online teaching and learning. For instance, research has found significant associations between privacy and faculty members’ intention to use cloud computing (Asadi et al., 2020), as well as students’ attitudes toward using cloud services (Arpaci et al., 2015) in educational settings. What’s more, students’ attitudes toward privacy influence the acceptance of learning analytic systems (Ifenthaler and Schumacher, 2016) and their online behaviors in higher education (Slade et al., 2019). Furthermore, this phenomenon may vary depending on the characteristics of the population. According to a study, individuals more concerned about their online privacy than others also held substantially more negative attitudes toward information sharing (Eurobarometer, 2015). In live online classes, some learners and educators may feel uncomfortable with a live-streamed camera being opened and exposing their home environments. A study has indicated that students retain a degree of privacy concerns regarding the accidental exposure of their home-lives during an online class (Selwood, 2021). Base on these observations, we propose the following hypothesis:

*H7*: Teachers’ private concerns play a moderating role on perceived ease of use and user satisfaction of live online teaching.

*H8*: Teachers’ private concerns play a moderating role on perceived usefulness and user satisfaction of live online teaching.

*H9*: Teachers’ private concerns play a moderating role on perceived ease of use and adoption of live online teaching.

**Figure 1:**
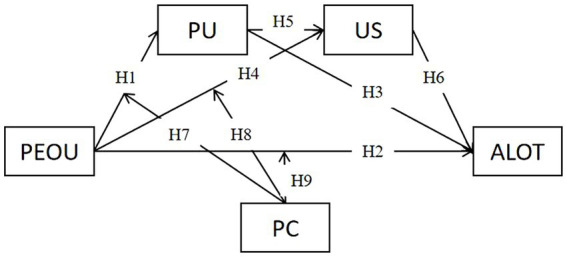
The proposed research model. PEOU, Perceived Easy of Use; PU, Perceived Usefulness; PC, Privacy Concerns; US, User Satisfaction; ALOT, Adoption of Live Online Teaching.

Based on the above theories and research hypothesis, we propose the following research model, see Figure 1.

## 3 Materials and methods

### 3.1 Procedures

This study explores teachers’ intentions toward adopting live online teaching in higher education. To achieve the study’s objective, we formulated a research model and conducted an empirical investigation at a university in eastern China. Participants were selected through a simple random sampling method. Specifically, we distributed an anonymous online survey to the faculty social media groups of each college within the university, allowing each teacher a single opportunity to respond to the survey. A brief introduction to the study was presented in both the faculty social media groups and at the beginning of the survey, and written consents were obtained from all individual participants engaged in the data collection process. After completing the survey, each participant was eligible for a cashback reward range from 1 to 5 CNY.

### 3.2 Participants

A total of 224 questionnaires were collected, of which 18 were deemed invalid due to irregular responses or other issues (ie., 7 samples provided the same answer for all questions, and 11 samples showed no live online teaching experience). Consequently, a final sample of 206 valid questionnaires was obtained, resulting in an effective response rate of 91.96%. As shown in Table 1, among the participants, 46.60% were male, and 53.40% were female. Regarding disciplines, 43.70% of participants were from Humanities and Social Sciences, 21.36% were from Natural Sciences, 29.12% were from Engineering, and 5.82% belonged to other disciplines. Regarding teaching experience, the distribution among the participants was as follows: 26.21% had 0–3 years of experience, 23.30% had 4–5 years, 19.42% had 6–10 years, 17.48% had 11–20 years, and 13.59% had over 20 years of teaching experience. The participants have varying levels of live online teaching experience, with 37.40% having 1–5 times, 15.00% having 6–10 times, 16.50% having 11–20 times, and 31.10% having more than 20 times.

**Table 1:** Basic information of the participants.

Basic information	Type	Percentage
Gender	Male	46.60%
	Female	53.40%
Discipline	Humanities and social sciences	43.70%
	Natural sciences	21.36%
	Engineering	29.12%
	Others	5.82%
Teaching experience	0–3 years	26.21%
	4–5 years	23.30%
	6–10 years	19.42%
	11–20 years	17.48%
	Over 20 years	13.59%
Live online teaching experience	1–5 times	37.40%
	6–10 times	15.00%
	11–20 times	16.50%
	Over 20 times	31.10%

### 3.3 Measures

Data were collected through a questionnaire comprising of two parts. The first section covered demographic data, including gender, discipline, teaching experience, and live online teaching experience. The second section included four scales to measure the studied variables. Items were rated on a 5-point Likert scale from 1 (totally disagree) to 5 (totally agree), with a higher score indicating a higher level of the variable.

The Technology Perception Scale is adapted from Davis (1989) within the context of live online teaching. Both perceived ease of use and perceived usefulness were measured with five items. A sample item for perceived ease of use could be “I think the live online teaching platform is easy to use,” and a sample item for perceived usefulness could be “I believe live online teaching can overcome time and spatial limitations to conduct flexible teaching.”

The User Satisfaction Scale is developed based on the system satisfaction scale proposed by Liaw et al. (2008). It measures teachers’ satisfaction with live online teaching using four items, such as “I am satisfied with the functions provided by the live online teaching platforms.”

The Privacy Concerns Scale adopts the information privacy concerns scale proposed by Xu et al. (2012). It measures teachers’ privacy concerns regarding live online teaching from three aspects: perceived surveillance, perceived intrusion, and secondary use of information. For example, the item related to perceived surveillance is “I worry that there will be other people watching during the live online teaching process, and I feel like I am being watched.”

The Adoption of Live Online Teaching Scale is adapted from the online learning system continuance use scale proposed by Lin and Wang (2012). It consists of four items to evaluate teachers’ adoption of live online teaching, such as “I intend to continue using the live online teaching platforms,” and “I will improve the use of live online teaching in my class next time” etc.

### 3.4 Data analysis

The data were processed and analyzed using SPSS 25.0, PROCESS 4.0, and AMOS 24.0. In detail, SPSS 25.0 was used for common method bias testing, reliability testing, exploratory factor analysis, descriptive statistical analysis, and correlation analysis. AMOS 24.0 was employed for confirmatory factor analysis to assess the scale’s validity. In addition, PROCESS 4.0 was applied to examine the theoretical model proposed in this study.

Initially, Harman’s one-way test was conducted to examine potential common method bias. Subsequently, the questionnaire underwent reliability and validity testing. Descriptive statistics were examined, and a correlation analysis of the variables was conducted. The research model was assessed through PROCESS 4.0 Model 85. Finally, specific moderation effects were explored using simple slope analysis. Bootstrap procedures were employed to examine the mediation and moderated mediation effects, with a bias-corrected bootstrapping (n = 5,000) with 90% confidence intervals (CI), the effects were considered significant when the confidence intervals did not include zero. Teachers’ gender, discipline, and teaching experience were included as control variables in the data analysis.

## 4 Results

### 4.1 Common method bias detection

To control for common method bias, an anonymous survey approach was applied during data collecting process. Subsequently, Harman’s one-way test was conducted to assess the collected data. The results showed that five factors had eigenvalues greater than 1 in exploratory factor analysis. Notably, the first factor, explaining 37.283% of the variance, which was lower than the critical standard of 50% recommended by Hair et al. (2019). This outcome suggested the absence of significant common method bias in this study.

### 4.2 Reliability and validity test

Firstly, exploratory factor analysis was conducted on the questionnaire using SPSS 25.0, resulting in the extraction of five factors. Each variable was accurately aligned with the corresponding measurement items. However, one item related to perceived usefulness (“I think live online teaching is convenient to watch again and again since it has the recording function”) exhibited factor loading and common factor variances below 0.5. As a result, this item was excluded from the data sample. Subsequently, a reliability test was performed using SPSS 25.0 (see Table 2), revealing an overall reliability of 0.879, with Cronbach’s α coefficient of each variable greater than 0.7. This indicating that the scale demonstrated high internal consistency. To assess validity, confirmatory factor analysis was carried out using AMOS 24.0. As we can see from Table 2, the standardized factor loading (FL) for each item exceeded 0.5, the composite reliability (CR) for each variable was greater 0.8, and the average variance extracted (AVE) of each variable was higher than 0.5. These results suggest that the convergent validity was ideal. Additionally, the structural model was fitted using AMOS, yielding the following fit indices: χ2/df = 1.759, GFI = 0.886, AGFI = 0.847, NFI = 0.901, IFI = 0.955, CFI = 0.954, and RMSEA = 0.061. Except for GFI, which approached the critical value of 0.9 (considered acceptable), all other indices met the recommended values, indicating that the structural model fitted well.

**Table 2:** Test of reliability and validity of measurement scales.

Study variables	Cronbach’s *α*	FL	CR	AVE
Perceived ease of use	0.865	0.639–0.850	0.859	0.553
Perceived usefulness	0.849	0.604–0.864	0.826	0.548
User satisfaction	0.882	0.663–0.893	0.879	0.649
Privacy concerns	0.826	0.761–0.814	0.827	0.615
Adoption of live online teaching	0.900	0.716–0.934	0.890	0.672

### 4.3 Descriptive statistics and correlation analysis

The study conducted descriptive statistics and correlation analysis on all studied variables. Table 3 presents the means and standard deviations of the variables along with the correlations between them. The results indicated that perceived ease of use was significantly positively correlated with perceived usefulness (*r* = 0.319, *p* < 0.001), user satisfaction (*r* = 0.425, p < 0.001), and adoption of live online teaching (*r* = 0.348, *p* < 0.001). Perceived usefulness exhibited significant positive correlations with user satisfaction (*r* = 0.632, *p* < 0.001) and adoption of live online teaching (*r* = 0.613, *p* < 0.001). User satisfaction showed a significant positive correlation with adoption of live online teaching (*r* = 0.645, *p* < 0.001). Privacy concerns were significantly negatively correlated with perceived ease of use (*r* = −0.233, *p* < 0.01). The square root of the average variance extracted (AVE) values for each variable (the bold value in the Table 3) exceeded the correlation coefficients between variables (the pairwise correlation coefficients contained in its submatrix). This observation indicated that the measurement scale demonstrated good discriminant validity.

**Table 3:** Descriptive statistics and correlation coefficients of study variables.

Study variables	*M*	SD	Perceived ease of use	Perceived usefulness	User satisfaction	Privacy concerns	Adoption of live online teaching
Perceived ease of use	4.040	0.724	**0.744**				
Perceived usefulness	3.158	0.872	0.319***	**0.740**			
User satisfaction	3.375	0.827	0.425***	0.632***	**0.805**		
Privacy concerns	2.769	0.974	−0.233***	−0.057	−0.039	**0.784**	
Adoption of live online teaching	3.450	0.898	0.348***	0.613***	0.645***	−0.057	**0.820**

***p < 0.001, **p < 0.01, *p < 0.05; M, Means; SD, standard deviations.

### 4.4 Proposed model test

We utilized Model 85 in Process 4.0 to explore the moderated mediation model proposed in this study (results are shown in Tables 4, 5). As indicated in Table 4, perceived ease of use had a significant direct effect on perceived usefulness (*β* = 0.370, *p* < 0.001) and user satisfaction (*β* = 0.290, *p* < 0.001), thereby supporting hypothesis H1 and H4. Perceived usefulness demonstrated a significant direct effect on user satisfaction (*β* = 0.513, *p* < 0.001) and adoption of live online teaching (*β* = 0.343, *p* < 0.001), supporting hypothesis H5 and H3. User satisfaction showed a significant direct effect on adoption of live online teaching (*β* = 0.425, *p* < 0.001), supporting hypothesis H6. However, perceived ease of use did not have a significant direct effect on adoption of live online teaching (*β* = 0.080, *p* = 0.267), rejecting hypothesis H2.

**Table 4:** Proposed model test.

Predictors	Model 1 (Outcome: PU)			Model 2 (Outcome: US)			Model 3 (Outcome: ALOT)		
	*β*	se	*t*	*β*	se	*t*	*β*	se	*t*
Constant	3.384	0.253	13.391***	1.786	0.258	6.912***	1.222	0.303	4.028***
Sex	−0.155	0.117	−1.328	−0.014	0.087	−0.157	−0.097	0.092	−1.050
Subject	−0.037	0.049	−0.750	0.018	0.037	0.483	−0.013	0.039	−0.328
Experience	0.041	0.042	0.966	−0.012	0.031	−0.388	−0.039	0.033	−1.175
PEOU	0.370	0.083	4.445***	0.290	0.065	4.481***	0.080	0.072	1.113
PC	−0.001	0.062	−0.010	0.034	0.046	0.736	−0.015	0.049	−0.303
PU				0.513	0.053	9.765***	0.343	0.067	5.080***
US							0.425	0.075	5.676***
PEOU*PC	0.131	0.072	1.833*	0.090	0.054	1.672*	0.065	0.057	1.144
R^2^	0.126			0.468			0.501		
*F*	4.776***			24.855***			24.764***		

***p < 0.001, **p < 0.01, *p < 0.1; Control variables: gender, subject, and teaching experience; PEOU, Perceived Easy of Use; PU, Perceived Usefulness; PC, Privacy Concerns; US, User Satisfaction; ALOT, Adoption of Live Online Teaching.

**Table 5:** Bootstrap test of conditional process.

Mediation path	Level of privacy concerns	Effect	Bootstrap SE	Bootstrap LLCI	Bootstrap ULCI
PEOU → PU → ALOT	Eff1 (PC = M-1SD)	0.083	0.042	0.016	0.152
	Eff2 (PC = M)	0.127	0.042	0.056	0.197
	Eff3 (PC = M + 1SD)	0.171	0.058	0.075	0.266
	Index of moderated mediation	0.045	0.029	0.006	0.097
PEOU → US → ALOT	Eff1 (PC = M-1SD)	0.086	0.039	0.020	0.148
	Eff2 (PC = M)	0.123	0.036	0.069	0.186
	Eff3 (PC = M + 1SD)	0.161	0.055	0.084	0.262
	Index of moderated mediation	0.038	0.032	0.001	0.103
PEOU → PU → US→ALOT	Eff1 (PC = M-1SD)	0.053	0.036	0.008	0.125
	Eff2 (PC = M)	0.081	0.035	0.035	0.148
	Eff3 (PC = M + 1SD)	0.109	0.039	0.054	0.181
	Index of moderated mediation	0.029	0.015	0.006	0.054

PEOU, Perceived Easy of Use; PU, Perceived Usefulness; PC, Privacy Concerns; US, User Satisfaction; ALOT, Adoption of Live Online Teaching.

According to Table 4, the interaction term between perceived ease of use and privacy concerns significantly influenced perceived usefulness (*β* = 0.131, *p* < 0.1), indicating that privacy concerns had a notable moderating effect on the relationship between perceived ease of use and perceived usefulness, thus supporting H7. Similarly, the interaction term between perceived ease of use and privacy concerns significantly affected user satisfaction (*β* = 0.090, *p* < 0.1), showing that perceived ease of use had a meaningful moderating effect on the connection between perceived ease of use and user satisfaction, in line with H8. Obviously, there was no significant moderating effect observed on the relationship between perceived ease of use and adoption of live online learning, leading to the rejection of H9.

**Figure 2:**
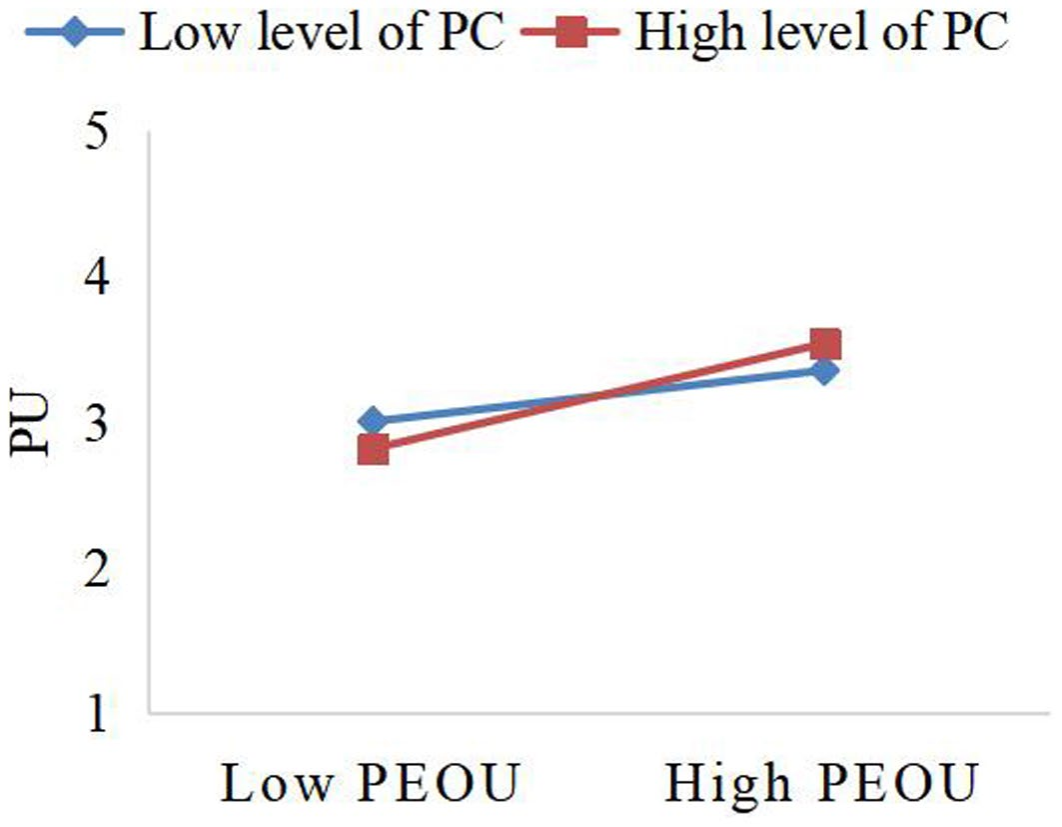
The moderating effect of PC between PEOU and PU. PEOU, Perceived Easy of Use; PU, Perceived Usefulness; PC, Privacy Concerns.

**Figure 3:**
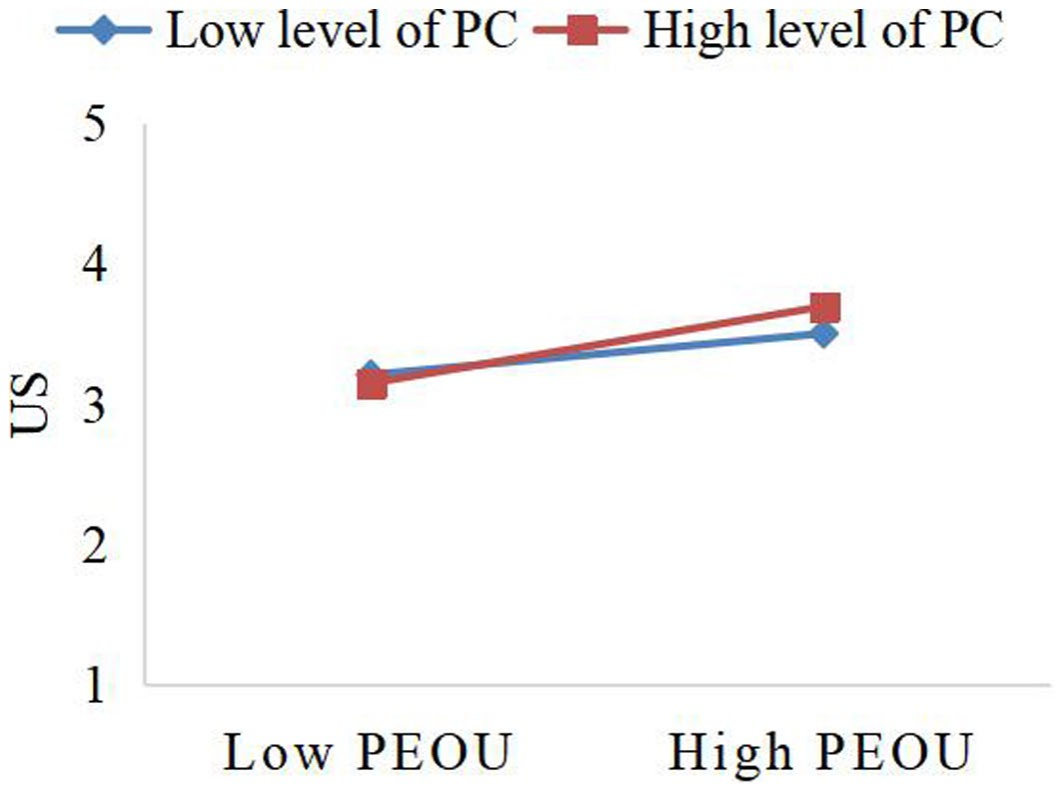
The moderating effect of PC between PEOU and US. PEOU, Perceived Easy of Use; PC, Privacy Concerns; US, User Satisfaction.

To understand the specific moderating effect of privacy concerns on the relationship between perceived ease of use and perceived usefulness, as well as perceived ease of use and usage satisfaction, a simple slope test was executed in this study (see Figures 2, 3). The results, depicted in Figures 2, 3, indicate that perceived ease of use positively predicts perceived usefulness and user satisfaction. Importantly, high privacy concerns enhance this impact, suggesting that privacy concerns serve as a booster in this process. Regarding the moderating effect, privacy concerns exhibit a relatively stronger moderating effect on the relationship between perceived ease of use and perceived usefulness (*β* = 0.131, *t* = 0.072, *p* < 0.1) compared to the relationship between perceived ease of use and user satisfaction (*β* = 0.090, *t* = 0.054, *p* < 0.1).

We employed the Bootstrap method to test the moderated mediation effects at a 90% confidence level, if the confidence interval does not include 0, then the moderated mediation is significant (Preacher and Hayes, 2008). To gain a deeper understanding of the moderating role in various mediation path, we compared the indirect effects at different levels of privacy concerns (M-1SD, M, and M + 1SD). As presented in Table 5, perceived usefulness played a mediating role between perceived easy of use and adoption of live online teaching, while this mediation effect was stronger for teachers with higher level of privacy concerns (conditional indirect effect = 0.171, 90% CI = [0.075, 0.266]) compared to teachers with low level of private concern (conditional indirect effect = 0.083, 90% CI = [0.016, 0.152]). Likewise, the index of moderated mediation was 0.038 with a 90% CI of [0.001, 0.103] and 0.029 with a 90% CI of [0.006, 0.054], indicating that privacy concerns strengthened the mediation effects of other two path showed in Table 5.

## 5 Discussion

### 5.1 Theoretical contributions

The impact of perceived ease of use and perceived usefulness on teachers’ adoption of live online teaching exhibits notable distinctions.

Drawing on the Technology Acceptance Model (TAM), technology perception, which including perceived ease of use and perceived usefulness, serves as a significant predictor of technology adoption. A substantial body of empirical research has consistently demonstrated the positive influence of perceived usefulness on technology acceptance (Venkatesh and Davis, 2000). However, empirical studies within the field of educational research have yielded conflicting findings regarding the relationship between perceived ease of use and technology acceptance. While Mazman Akar (2019) identified perceived ease of use as a direct determinant of teachers’ technology acceptance, other studies, including Nagy (2018), reported that perceived ease of use has no significant direct effect on technology acceptance. This study aligns with the latter perspective, which means perceived ease of use has no significant direct effect on the adoption of live online teaching. This outcome may be attributed to the combined effect of the low entry barriers for utilizing live online teaching platforms and the high level of information technology literacy among university teachers. The contemporary live online teaching platforms offer user-friendly interfaces with low entry barriers, coupled with the proficient information technology literacy among most university teachers, the perceived ease of use of live online teaching platforms appears to have a minimal impact on their adoption.

User satisfaction is an important mediator in the relationship between technology perception and teachers’ adoption of live online teaching.

It is believed that teachers’ satisfaction with teaching technology plays a crucial role in their willingness to continue using it (Chiu et al., 2007). Factors influencing user satisfaction have been found to impact teachers’ willingness to continue using technology through changes in their satisfaction levels (Yeung and Jordan, 2007). Young (2013) discovered that students’ perceived usefulness and perceived ease of use of MOOCs positively influence their willingness to continue using these platforms, with user satisfaction acting as a mediating factor in this process. This study’s findings align with the existing empirical research, indicating that teachers’ satisfaction serves as a significant mediating factor between their perception of technology and their adoption of live online teaching.

Privacy concerns exhibit a moderating impact on teachers’ adoption of live online teaching, and an interesting phenomenon known as the “privacy paradox” has been observed.

This study reveals that privacy concerns have a moderating effect on the three mediating paths within the research model. Specifically, teachers with a higher level of privacy concerns are more satisfied with live online teaching and more inclined to adopt it. Such a conclusion may appear counterintuitive based on prevalent assumptions. Typically, individuals with high level of privacy concerns are thought to have a heightened awareness of privacy risks associated with the media technology use. This heightened awareness can induce anxiety about privacy risks, potentially affecting the overall media use experience and the willingness to continue using it (Xu et al., 2012). However, some studies have uncovered a paradoxical phenomenon known as the “privacy paradox,” wherein users exhibit active information disclosure despite possessing high levels of privacy concerns. This inconsistency between privacy attitudes and behaviors can be influenced by various factors such as media trust (Aguirre et al., 2016) and social rewards (Jiang et al., 2013). The findings of this study reflect the presence of the privacy paradox in the context of live online teaching. The authors believe that this phenomenon is primarily associated with teachers’ consideration of “expected benefits-privacy risks” and their privacy self-efficacy when engaging in live online teaching. Aligned with the privacy calculus theory, individuals tend to derive satisfaction from media use when they perceive the benefits to outweigh the associated privacy risks (Baruh et al., 2017). Specifically, when users believe they have a reasonable degree of control over privacy risks, their confidence and sense of accomplishment in managing privacy further increase (Chen and Chen, 2015). Consequently, individuals with high level of privacy concerns are more likely to experience the satisfaction, leading to an enhancement of their adoption of media use. Overall, although teachers with high level of privacy concerns may be more prone to worry about privacy threats in live online teaching, they are more likely to embrace it because they perceive it as a beneficial instructional tool and consider the privacy threats to be manageable through strategies such as background blocking, video and microphone adjustment, and other relevant measures. This nuanced perspective sheds light on the complex interplay between privacy concerns and teachers’ adoption of live online teaching.

### 5.2 Practical implications

Inspiring media corporations to take social responsibility and optimizing the design of live online teaching platforms.

The study reveals that perceived ease of use has a positive impact on both perceived usefulness and user satisfaction. Furthermore, perceived usefulness and user satisfaction directly influence teachers’ adoption of live online teaching. These findings highlight the importance of enhancing the design and development of live online teaching platforms to ensure their efficacy and user-friendliness. Despite the abundance of live online teaching platforms offering practical functionalities, there is a call for further optimization in platform design. Tailoring these platforms to meet specific teaching requirements and providing teachers personalized, user-friendly, and intelligent teaching support services is crucial. For instance, integrating “self-service” classroom management functions, such as attendance tracking, random roll call, and automatic queue management, can enhance interpersonal interaction and improve the quality of real-time engagement. Additionally, the incorporation of intelligent “teaching assistants” and “learning companions” with virtual avatars can facilitate human-computer interaction and provide supportive companionship throughout the teaching process, thereby alleviating the potential feelings of awkwardness and loneliness for both teachers and students when facing screens individually. Furthermore, although teachers serve as the designers and organizers of live online teaching activities, their roles as “anchors” also require feedback and motivation from students. To foster emotional communication, live online teaching platform can integrate personalized mechanisms, allowing interactive features during specific segments. This empowers students to express themselves through various forms of interactivity, such as likes, virtual gifts, applause, and emojis, fostering an environment conducive to emotional communication. This dual focus on refining platform features and encouraging emotional engagement contributes to an enriched live online teaching experience.

Encouraging educational management departments to support teachers’ information literacy and professional development.

This study discovers the presence of a “privacy paradox” phenomenon in teachers’ adoption of live online teaching. This paradox is rooted in teachers’ considerations of the balance between “expected benefits” and “privacy risks,” as well as their confidence in managing privacy challenges. Specifically, the findings indicate that if teachers have a high level of confidence in their ability to manage privacy risks, they may engage in live online teaching more actively despite having significant privacy concerns. Therefore, university management departments should collaborate with media publicity departments to provide comprehensive and adaptable technical support services for teachers. According to a research, 63.5% of teachers feel the need for training or guidelines in using online teaching platforms (Hassan et al., 2020). This can be accomplished through various means such as training courses, demonstration videos, and user manuals, all geared toward bridging the technological divide. Moreover, it is crucial to establish specialized technical support departments that promptly address personalized usage issues. Furthermore, live online teaching requires a shift from replicating offline classrooms to targeted instructional design that considers the technological landscape and unique characteristics of student in live online teaching environment. Therefore, university teacher management departments should enhance teachers’ training in information technology instructional design. This can be achieved through dedicated research projects focused on live online teaching, enabling teachers to comprehend the distinct aspects of learning in live online teaching environment and facilitating a seamless transition in their roles as live online instructors. Conducting live online teaching seminars and specialized activities that discussing challenges encountered in practice can further enhance teachers’ instructional design capabilities. Additionally, educational management departments and relevant educational associations can organize activities to collect exemplary case studies of live online teaching across various educational stages and subjects. This initiative fosters mutual learning and self-improvement among peers by facilitating the exchange of experiences and insights. Overall, a proactive approach to supporting teachers in information literacy and professional development is vital for navigating the evolving landscape of live online teaching.

### 5.3 Limitations and future directions

While this study has provided valuable insights and implications, it is important to acknowledge certain limitations and suggest potential directions for future research. Firstly, the participants were recruited from a university in eastern China, which may limit the generalizability of the findings to other cultural and institutional contexts. Future research should enhance external validity by considering a more diverse sample such as including participants from other universities in China or even abroad. Additionally, the research focused on college teachers, and the findings may not be directly applicable to teachers at different educational levels. The dynamics of live online teaching could vary across educational settings, and future studies might investigate these variations for a more nuanced understanding. Transparently acknowledging these limitations aims to provide readers with a comprehensive understanding of the study’ s scope and encourages future research to address these constraints for a more thorough examination of teachers’ attitudes and continuance intention toward live online teaching.

## 6 Conclusion

This study has yielded several significant findings: (a) perceived easy of use has a positive impact on perceived usefulness and user satisfaction; (b) perceived usefulness has a positive effect on user satisfaction; (c) both perceived usefulness and user satisfaction positively influence teachers’ adoption of live online teaching; (d) perceived easy of use does not directly affect teachers’ adoption of live online teaching; (e) privacy concerns play a moderating role in the relationship between perceived easy of use and perceived usefulness, as well as the relationship between perceived easy of use and user satisfaction. These findings contribute to the development of a conditional process model, with perceived ease of use as a predictor, perceived usefulness and user satisfaction act as mediators, and privacy concerns function as a moderator. The findings suggest that stakeholders should collaborate closely to enhance the design and development of live online teaching platforms. Additionally, efforts should be made to improve teachers’ information literacy, thereby fostering their enthusiasm and facilitating their professional development in the practice of live online teaching.

## Data availability statement

The original contributions presented in the study are included in the article/supplementary material, further inquiries can be directed to the corresponding author.

## Ethics statement

The studies involving humans were approved by Ethics Committee in the College of Education Science and Technology at Nanjing University of Posts and Telecommunications. The studies were conducted in accordance with the local legislation and institutional requirements. The participants provided their written informed consent to participate in this study.

## Author contributions

WX: Writing – original draft, Writing – review & editing, Conceptualization, Data curation, Formal analysis, Funding acquisition, Investigation, Methodology, Project administration, Resources, Software, Supervision, Validation, Visualization. MW: Data curation, Investigation, Writing – review & editing. JM: Data curation, Investigation, Writing – review & editing.
